# The Compositional HJ-Biplot—A New Approach to Identifying the Links among Bioactive Compounds of Tomatoes

**DOI:** 10.3390/ijms17111828

**Published:** 2016-11-02

**Authors:** Marcos Hernández Suárez, Daniel Molina Pérez, Elena M. Rodríguez-Rodríguez, Carlos Díaz Romero, Francisco Espinosa Borreguero, Purificación Galindo-Villardón

**Affiliations:** 1 Aula Dei Scientific Technological Park Foundation, Av. Montañana 930, 50059 Zaragoza, Spain; marcoshsuarez@gmail.com; 2 Department of Chemical Engineering and Pharmaceutical Technology, Food Science and Nutrition Area, University of La Laguna, 38206 La Laguna, Santa Cruz de Tenerife, Spain; dmolina.perez@gmail.com (D.M.P.); emrguez@ull.es (E.M.R.-R.); cdiaz@ull.es (C.D.R.); 3 Plant Biology, Ecology and Earth Sciences Department, Extremadura University, Av. Elvas s/n, 06071 Badajoz, Spain; 4 Statistics Department, University of Salamanca, c/Alfonso X El Sabio s/n, 37008 Salamanca, Spain; pgalindo@usal.es; 5 Higher Polytechnic School of the Litoral, Campus Gustavo Galindo, 09-01-5863 Guayaquil, Ecuador

**Keywords:** tomato, link, compositional Mann–Whitney U test, compositional HJ-biplot

## Abstract

Tomatoes have been described as a functional food because of their particular composition of different bioactive compounds. In this study, the proximate composition, minerals and trace elements, and antioxidant compounds were determined in two tomato cultivars (Mariana and Dunkan) that were grown in Gran Canaria (Spain) either conventionally or hydroponically. Although compositional data of this type require being subjected to the specific statistical techniques of compositional analysis, this approach has not usually been considered in this context. In the present case, a compositional Mann–Whitney U test of the data showed significant differences for each factor (cultivar and cultivation system) in several of the compositional variables studied. For the differences between cultivars, these parameters were the protein, Mg, lycopene, ascorbic acid, citric acid, and fumaric acid contents. For the differences between cultivation systems, they were mainly those of the mineral and trace elements group. Although one-year data are insufficient to make clear relationship among compounds because more repetitions in several localities and years are necessary, the compositional HJ-biplot (in which the links provide estimates of the linear relationship among variables) results agreed with other scientific results about linear relationship among some compounds analyzed.

## 1. Introduction

The tomato is one of the world’s most consumed vegetables and fruits. It is regarded as a functional food since the chemical composition of tomato samples has been found to include such bioactive constituents as fiber, minerals, vitamins, and phenolic and carotenoid compounds [1]. The Canary Islands are a major producer of tomatoes, with many of the cultivars used having particular and interesting characteristics [2]. Various factors are known to affect the nutrient content, including cultivar, climate, geochemistry, and agricultural practices. Hence the numerous studies aimed at evaluating and improving the quality of the tomato fruit [1]. However, the conclusions of those studies have been based on the application of standard statistical tools, while it has not been usual to consider this kind of data from the mathematical viewpoint of compositional analysis.

Compositional data are vectors of positive values that sum to unity (or, in general, to some constant that is fixed for all the vectors) [3]. In the present study, each tomato sample was defined by 25 chemical content variables whose sum accounts for almost 100% of the sample’s chemical composition. In the case of compositional data, the information that standard descriptive statistics can contribute is limited. The problem is that arithmetic means and variances are clearly defined in the framework of a Euclidean geometry, but this geometry is not appropriate for compositional data because the central tendency of a compositional sample is best represented by the geometric mean instead of the arithmetic mean, and this has consequences if comparisons are made using standard statistical tools based on this latter class of mean. The overall dispersion of a compositional sample is not the standard deviation but the total variance, given by the mean squared Aitchison distance [4].

**Figure 1:**
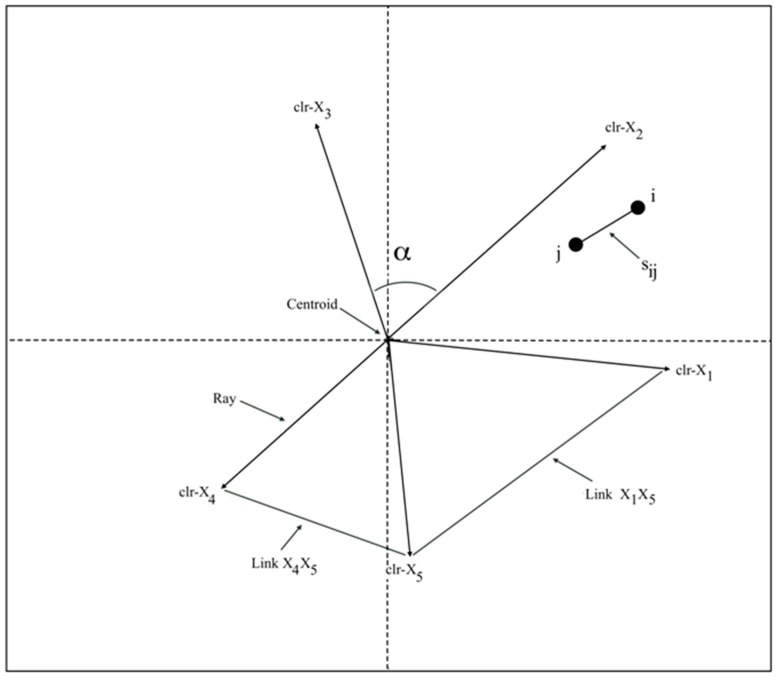
Elements of a compositional biplot.

The measure of correlation can also be quite misleading with compositional data. This phenomenon is known as “spurious correlation”, i.e., a false presumption that two variables are correlated when in reality they are not. This has consequences when a standard multivariate analysis based on a correlation matrix is applied to this kind of data [3]. Compositional biplots are graphical techniques that allow the visual identification of real associations between variables in this type of data. They were introduced by Aitchison and Greenacre [5] who proposed the terms “form biplot” and “covariance biplot” as equivalents for Gabriel’s “JK-biplot” and “GH-biplot”, respectively [6]. Such compositional biplots consist of vertices, rays, links, and markers related to each individual observation (Figure 1). A ray provides an estimate of the standard deviation of a variable, while a link is a measure of the strength of the linear relationship between two components of a composition. Links are not represented as arrows in the biplot. Unlike other biplots, in a compositional biplot, the links are the most relevant elements.

In plant physiology and plant nutrition, it is common to study relationships between variables. Examples are the Ca/Mg ratio for the preparation of balanced nutrient solutions, the Ca/oxalic acid ratio in order to analyze the bioavailability of Ca [7], and the Deviation from Optimum Percentage (DOP) index, expressed as DOP = C/Cref where C is the nutrient concentration in the sample to assess and Cref is the optimal nutrient concentration used as reference value, for information on nutritional status [8]. The link concept can be regarded as a statistical tool with which to study those and other relationships.

The purpose of this paper is to identify the link (and rays) among bioactive compounds of tomatoes through compositional HJ-biplot as alternative to the GH and JK compositional biplots. HJ-biplot was selected because it provides a higher goodness-of-fit than the Gabriel’s [9,10,11] for rows and columns of the matrix. Besides, the HJ-biplot is line with the prerequisite of singular value decomposition (SVD) of the original log-transformed data matrix indicated by Aitchison and Greenacre [5].

It would be interesting to extend this idea to other Biplots (Canonical biplot for example) but the impact on links should be carefully studied since working with the matrix centroids and not the original data matrix.

Prior to constructing the compositional HJ-biplot, we applied a compositional Mann–Whitney U-test to the data to assess the effect of cultivar and cultivation system on the overall chemical composition of the tomatoes. Both of these procedures require a log transformation to be performed [3,5].

## 2. Results and Discussion

### 2.1. General Description of the Tomato Samples

Table 1 and Table 2 give the results of the influence of cultivar and cultivation system on the chemical composition of the tomatoes (proximate composition, mineral elements, organic acids, and antioxidant compounds) together with the compositional Mann–Whitney U test results after centered log-ratio (clr)-transformation.

Moisture and ash showed no significant differences between cultivar or cultivation system. However, protein content, while similar to that reported by Valdivia-Mares et al. [12], was higher in cv. Mariana than in cv. Dunkan, and in the no-soil than in the soil condition. There were no differences in glucose or fructose content whose values were similar to the maximum values reported by Figàs et al. [13] and Lahoz et al. [14]. While fruit and vegetable mineral element concentrations are strongly influenced by the content of those elements in the soil or nutritional solution [15], no significant differences were found in total Fe, Cu, or Zn. However, P, Na, K, Ca, and Mn differed significantly by cultivation system, and Mg by both cultivar and cultivation system. Compared with the results of Erba et al. [16], our P, Ca, and Mg values were greater, our Cu and Zn lower, and the rest of the mineral elements similar. The lycopene, ascorbic acid, and phenol contents were similar to the low values reported by Figàs et al. [13]. For citric acid, the contents were higher than those reported by Lahoz et al. [14], while for malic acid they were lower.

Considering just the effect of the tomato cultivar, the cv. Mariana’s content was higher in protein, Mn, and ascorbic acid, and lower in Mg, lycopene, and citric and fumaric acids than cv. Dunkan’s.

Considering the cultivation system, the main differences were in the group of mineral and trace elements. For the soil-grown tomatoes, the Ca, Mn, and phenol contents were higher, and the protein, P, Na, K, and Mg lower than in the no-soil tomatoes.

Tomato consumption is associated with healthy diets for its contribution to the daily intake of carotenoids (providing an estimated 80% of daily intake of lycopene) and of folate, ascorbic acid, flavonoids, α-tocopherol, and potassium [17,18]. However, other nutritionally important variables such as the oxalic acid content must also be considered. This dicarboxylic acid forms insoluble salts with calcium and other divalent cations, leading to a decrease in their bioavailability. It is therefore considered to be an anti-nutrient compound inhibiting the correct absorption of Ca [7]. According to Mitjavila [19], 2.5 g of oxalic acid precipitates with 1 g of Ca, so that its anti-nutrient effect is determined by the oxalic acid/Ca ratio. According to the values given in Table 1 and Table 2, for cv. Dunkan, this ratio was 1.90 and 2.08 for the soil and no-soil cultivation systems, respectively, while for cv. Mariana, the ratio was lower, 1.54 and 1.72, respectively. These differences were not found to be significant, however.

### 2.2. Differences in Bioactive Compounds by Cultivar and Cultivation System

The antioxidant profile of the diet has recently been recognized as being an important variables for predicting the impact of food on human health [20]. The main antioxidant of tomato, lycopene, has been widely reported to play a major role in the prevention of cardiovascular disease and various types of cancer [21]. One must also consider the content and relationships of phenolics (including chlorogenic, caffeic, and ferulic acids) and other bioactive compounds such as ascorbic acid, K, and Mg.

In the context of compositional statistics, the analysis of such relationships requires the use of logarithms of the ratios between variables, i.e., between the parts of the compositional vector. When these log-ratios are taken to be those between each component and the geometric mean, they are known as centred log-ratios [5,22].

**Figure 2:**
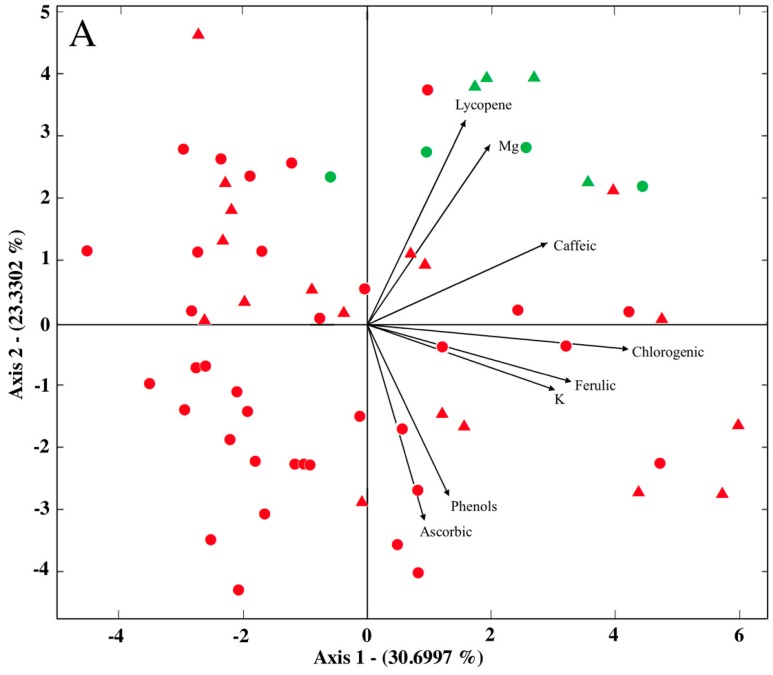

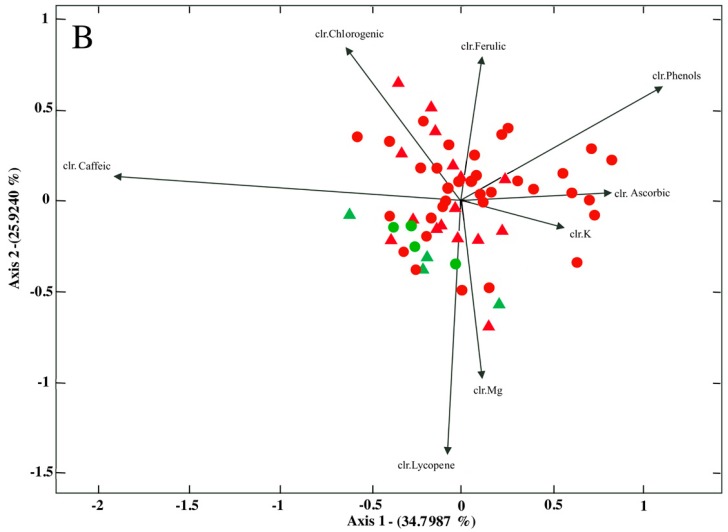
(**A**) Standard HJ-biplot; and (**B**) Compositional HJ-biplot of the chemical composition data. The Mariana tomato cultivar is represented in red; the Dunkan in green. The soil cultivation method is represented with the marker ●; the no-soil with the marker ▲.

It is important to emphasize how the problem of spurious correlations is resolved with compositional statistics. By way of illustration, a standard HJ-biplot (Figure 2A) and a compositional HJ-biplot (Figure 2B) were constructed for lycopene, total phenolics, chlorogenic acid, caffeic acid, ferulic acid, ascorbic acid, K, and Mg. The HJ-biplot was constructed with data expressed as percentages, and the compositional HJ-biplot with centred log-ratios, in both case with a double-centring transformation of the matrix. The angles between the vectors approximate the correlations between variables in such a way that small acute angles are associated with variables that are strongly positively correlated, obtuse angles close to 180° with variables that are strongly negatively correlated, and right angles with uncorrelated variables. One observes different correlations between the variables, and different percentages of the variance explained in each biplot, with an overall fit of 54.00% in the HJ-biplot and 60.72% in the compositional HJ-biplot.

Regarding compositional HJ-biplot (Figure 2B), samples near the centroid have values similar to the geometric mean for each component, while those far from the center can be diagnosed as having a large relative variation with respect to the geometric mean for the clr-transformed chemical variables that the points are near. In the case of the rays, caffeic acid has the greatest variation with respect to its mean, while the smallest variation corresponds to K. One observes a tendency that distinguishes between the two cultivars. The cv. Mariana samples are mainly located near the rays of chlorogenic, ferulic, and ascorbic acids, phenolics, and K, while the cv. Dunkan samples are near the rays of lycopene, caffeic acid, and Mg. Indeed, the highest values for each of those chemical variables correspond to the respective cultivars (Table 1 and Table 2). Considering just the no-soil samples in particular, the main effect of the cultivar is in the greater content of chlorogenic and ferulic acids for cv. Mariana, and of lycopene, caffeic acid, and Mg for cv. Dunkan.

All these relationships required more detailed consideration, especially that of lycopene with the rest of the variables. The acute angles for Mg/lycopene and K/ascorbic acid indicate positive correlations, a correlation that is especially strong in the case of Mg. This is coherent with the shortness of the Mg/lycopene link (see the rules for interpretation described in the Statistical Methods Section). In order to quantify the relationship, the values of the variance given in the upper triangle of Table 3 must be considered. For the Mg/lycopene log-ratio, the variance corresponds to a standard deviation of 0.207. Since this value is expressed on a logarithmic scale, it must be back-converted to a Euclidean scale, i.e., e0.207 ≈ 1.23. Thus, an almost linear increase in the lycopene content is due to the Mg content of the tomato fruit. However, K is more strongly correlated with the ascorbic acid than the lycopene content, with an estimated standard deviation of 1.14 (K/ascorbic acid). Fanasca et al. [23] showed that the cations K, Ca, and Mg affect the antioxidant content of tomatoes, especially under hydroponic cultivation. In our study too, the highest values of Mg and K were in the samples grown under no-soil conditions. In the case of lycopene/ferulic acid, the obtuse, close to 180°, angle between their rays indicates a possible strong negative correlation. This could be because the antioxidant content depends on various factors. One such factor is ripening stage. Lycopene accumulates mainly in the final period of ripening, but the hydroxycinnamic acids such as ferulic acid do not [24]. Right angles mean uncorrelated variables, which here are lycopene/caffeic acid and lycopene/ascorbic acid. For the rest of the bioactive compounds, Figure 2 shows there to be a possible one-dimensional variability of chlorogenic acid, ferulic acid, and phenols (collinear vertices or parallel links). In addition, the parallelogram formed by chlorogenic acid, ferulic acid, phenols, caffeic acid, and ascorbic acid reveals a simple constant log-contrast [5]. This might be an indication that this group of antioxidants vary as a whole, independently of the lycopene content. Cle et al. [25] showed that ascorbic acid and some phenolics tend to accumulate from the green to the midripe stage, whereas the total carotenes increase constantly during the ripening process. In contrast, chlorogenic acid decreases during ripening.

## 3. Materials and Methods

### 3.1. Tomato Sampling

A total of 61 tomato samples were provided by the COAGRISAN company (Sociedad Cooperativa Agrícola de San Nicolás de Tolentino de Gran Canaria, Spain) from their trials field in Gran Canaria (Canary Islands, Spain). The fields were placed at 200 m above sea level. In particular, samples of approximately 1 kg were collected at the same degree of ripeness according to the points 7–8 of the Dutch “kleurstadia” tomato-colour scale. They belonged to two cultivars (Mariana and Dunkan) grown under two agricultural practices: soil, and no-soil on coconut fibre substrate. In all cases, the plants were grown under greenhouse conditions, pruned to double stems, and staked. All tomato samples were harvested at the same time. According to the seed company, both tomato cultivars are resistant to ToMV (tomato mosaic virus), *Fusarium oxysporum* f.sp. *lycopersici*, *Leveillula taurica*, TYLCV (tomato yellow leaf curl virus), and the Mariana cultivar is also resistant to *Verticillium*.

In those trials, the UNE 155102 quality certification standard for the controlled production of tomatoes was followed, as were various European regulations on organic production and maximum pesticide residue levels. For the no-soil tomatoes, the nutrient solution consisted of 12 mM NO_3_^−^, 0.5 mM NH_4_^+^, 1.6 mM H_2_PO_4_^−^, 7 mM K^+^, 4.5 mM Ca^2+^, 3.6 mM Mg^2+^, 2.0 mM SO_4_^2−^, 5 µM Fe-EDTA, 2 µM Mn, 1 µM Zn, 50 µM B, 0.25 µM Cu, and 0.1 µM Mo. Its pH was 5.6–6.0. For the soil tomatoes, the nutrient requirements were adjusted to 690 kg/ha N, 460 kg/ha P_2_O_5_, 1714 kg/ha K_2_O, 1135 kg/ha CaO, 231 kg/ha MgO and 1797 kg/ha SO_3_, yearly.

### 3.2. Sample Preparation

Each sample was weighed, and the weight was divided by the number of tomatoes to give the weight per tomato. Then, three tomatoes were selected at random from each sample for analysis. They were hand-rinsed with ultra-pure water, shaken to remove any excess water, and gently blotted with a paper towel. A small portion (approximately 3 g weight) of each tomato was cut and put into 10 mL of 3% metaphosphoric acid as subsamples for the ascorbic acid assay. The tomatoes were then mixed and homogenized to homogeneous purée. A fraction of this purée was desiccated at 105 °C, homogenized again, and stored in a polyethylene tube at room temperature until assay for metals and protein. The rest was immediately stored in a polyethylene tube at −80 °C in order to avoid enzymatic changes and for the measurement of the other chemical variables.

### 3.3. Analytical Methods

All assays were performed in triplicate, and the results expressed per fresh weight (FW). Moisture was determined using the oven-drying method [26]. The residue was heated at 550 °C for 24 h for the ash determination [26]. Organic nitrogen was determined by the Kjeldahl method [26], and the protein concentration estimated using 6.25 as the conversion factor. The method of Kujala et al. [27] was used to determine the phenolic compound content: after the colorimetric reaction with the Folin-Ciocalteu reagent (Sigma-Aldrich Chemical Co., St. Louis, MO, USA), absorbance was measured at 750 nm using gallic acid (Sigma-Aldrich) standard solutions for the external standard calibration curve. Ascorbic acid was determined in the individual tomatoes using the 2,6-dichlorophenolindophenol titration procedure [26]. The lycopene concentration was determined spectrophotometrically at 503 nm following extraction in 20 mL of acetone:ethanol:hexane (5:5:10 *v*/*v*) in the dark, in accordance with the method described by Fish et al. [28].

Mineral and trace elements were determined by atomic absorption spectrometry using a Varian SpectrAA-10Plus (Varian Iberica SL, Madrid, Spain) atomic absorption spectrometer equipped with a deuterium-lamp background correction system using an air-acetylene flame. The dried tomato samples were previously acid-digested in nitric acid, in accordance with the procedure described by Hernández Suárez et al. [9].

Sugars were determined as described by Rodríguez Galdón et al. [29]. About 1 g of the frozen homogenized tomato was weighed directly in polypropylene tubes and mixed with ethanol:water (80:20 *v*/*v*). One milliliter of the solution was filtered (0.45 μm filter GHP, Waters, Milford, MA, USA), and the sugars were assayed by high-performance liquid chromatography (HPLC) using a Waters 2690 chromatograph equipped with a Waters model 2414 differential refractive index (DRI) detector (Waters, Milford, MA, USA). The separation was performed using a Waters carbohydrate analysis column (3.9 mm × 300 mm i.d.) with a particle-size diameter of 10 mm, equipped with a Waters CarboTM4-mm carbohydrate guard column. The eluent was acetonitrile:water (80:20 *v*/*v*), and the peaks were identified by comparison with the retention times of standard compounds.

Organic acids were determined by HPLC as described by Hernández Suárez et al. [30]. About 1 g of the frozen homogenized tomato was weighed directly in polypropylene tubes and mixed with ethanol:water (80:20 *v*/*v*). After filtering, the liquid phase was concentrated with a nitrogen stream, and the residue was redissolved in ultrapure water (Milli-Q water system, Waters, Milford, MA, USA), followed by filtration through a 0.45 μm pore size membrane and a Sep-Pak Accell Plus QMA cartridge (Waters, Milford, MA, USA) which had been pre-conditioned with 3 mL of ultrapure water. The eluent used was 2 mL of 20 mM sodium dihydrogen phosphate (adjusted to pH 1.0 with phosphoric acid buffer). The analytical system was a Waters 2690 high-performance liquid chromatograph equipped with a Waters 996 photodiode array detector (Waters, Milford, MA, USA). Separation was performed using a Waters Atlantis dC18 steel column (150 mm × 4.6 mm i.d.) with a particle diameter of 3 μm equipped with a Waters Atlantis dC18 guard column (20 mm × 4.6 mm). The eluent was 20 mM sodium dihydrogen phosphate (adjusted to pH 2.7 with phosphoric acid buffer), and the 210-nm wavelength peaks were identified by comparison with the retention times of standard compounds.

Free hydroxycinnamic acids were determined by HPLC as described by Hernández Suárez et al. [31]. About 1 g of the frozen homogenized tomato was mixed with 75% methanol (pH 2.5 adjusted with trifluoroacetic acid (TFA)), heated to 40 °C for 30 min, and then centrifuged. The supernatant was filtered (0.45 μm pore size) prior to HPLC analysis. The analytical system was the same as that used for the organic acid assays. The separation was performed using a Nova-Pak C18 steel cartridge (150 mm × 3.9 mm i.d.) with a particle diameter of 4 μm equipped with a Waters C18 guard column. The eluent was 0.05% TFA (pH 2.0) and methanol. For the detection, three wavelengths were used (280, 290, and 329 nm).

### 3.4. Statistical Methods

In order to apply standard statistical multivariate analysis, the raw compositional data were subjected to a clr-transformation. This transformation is symmetric with respect to the compositional parts, and maintains the same number of components as the number of parts in the composition. Its advantage is that it is an isometric transformation of the simplex with Aitchison metric onto a subspace of real space with the ordinary Euclidean metric [3,5,22]. The clr-transformation is given by the expression:

$$clr\left(x\right)=\left[ln\frac{x_{1}}{g_{m}\left(x\right)},\;ln\frac{x_{2}}{g_{m}\left(x\right)},\ldots ,\;ln\frac{x_{D}}{g_{m}\left(x\right)}\right]$$

where *x* = (*x*_1_, *x*_2_,…, *x_D_*) is a compositional data vector, the xi are the chemical variables all of which must be expressed in the same units, and *g_m_*(*x*) is the geometric mean of that compositional data vector. Calculations were performed using the CoDaPack 2.01 software package [32].

The sample sizes in the present study were unbalanced 8 tomato Dunkan cultivar tomato samples and 53 Mariana tomato cultivar samples from Gran Canaria (Spain) grown either conventionally or hydroponically. To compare the means of the variables, a Mann–Whitney U test was applied on the clr-transformed data (i.e., a compositional Mann–Whitney U test), with the significance level for differences among them being taken as *p* < 0.05. This calculation was performed using the SPSS vn. 21.0 program package (SPSS Inc., Chicago, IL, USA). The HJ-biplot is a joint representation in a low-dimensional vector space (usually a plane) of the rows and columns of a data matrix X, using markers (points/vectors) j_1_, j_2_, …, j_n_ for its rows and h_1_, h_2_, …, h_p_ for its columns. The markers are obtained from the usual singular value decomposition (SVD) of the data matrix X = UΣV^T^, where U is formed by the eigenvectors of the matrix XX^T^, V by the eigenvectors of the matrix X^T^X, and Σ is a diagonal matrix containing the singular values (i.e., the square roots of the non-zero eigenvalues of both XX^T^ and X^T^X), taking as rows the marker rows of J = UΣ and as columns the marker rows of H = VΣ, in the appropriate dimensions. Thus, the matrix X is formed by clr-transformed data, and then double-centered through its SVD to ensure that the components are analyzed on a ratio scale [5] in the appropriate dimensions.

A biplot of compositional data consists of the elements shown in Figure 1 [4,5]:

The similarity (Sij) between two samples or individuals is taken to be an inverse function of their distance, in such a way that closer samples are more similar.
The centroid represents the center-of-gravity formed by the geometric mean of the compositional parts used in the clr-transformation.
ray provides information on the variance of the corresponding log-ratio with respect to the geometric mean (gm):
  $$var\left(ln\frac{X_{i}}{g_{m}\left(X\right)}\right)$$

The square root of this expression is the standard deviation of the clr-transformed variable *X_i_*, and is represented by the length of the ray. The cosine of the angle (α) between two rays represents the approximate correlation coefficient between the corresponding variables.

The length of a link between the vertices of two rays represents the standard deviation of the log-ratio between the associated compositional parts, with the corresponding variance being defined as:

$$var\left(ln\frac{X_{i}}{X_{j}}\right)$$

Angles between links provide information on the relationships between pairs of variables. If two links intersect at a right angle then this is an indication that the pairs of variables are possibly uncorrelated (in Figure 1, links *X*_1_*X*_5_ and *X*_4_*X*_5_), while if they are parallel (or the angle is obtuse) then the pairs of parameters may be strongly correlated (in Figure 1, links *X*_1_*X*_5_ and *X*_4_*X*_3_). Coincident vertices or short links mean that the two variables are linearly proportional, so that the two parts involved can be assumed to be redundant. If a subset of links is collinear, this might indicate a possible one-dimensional variability.

The compositional HJ-biplot analyses were done using the MULTBIPLOT program package (MULTivariate analysis using BIPLOT) developed by Vicente-Villardón [33].

## 4. Conclusions

Compositional Data Analysis (CoDA) refers to the analysis of data, which have been defined as random vectors with strictly positive components whose sum is constant. In this paper we propose a new tool to analyze and represent compositional data, “Compositional HJ-biplot”, based on HJ-biplot and Aitchison Geometry to capture the algebraic-geometric structure of the data. Besides, this is the first time that an HJ-biplot multivariate analysis has been applied to chemical compositional data. Taking into account that HJ-biplot provides a better quality of representation than Gabriel biplots (GH and JK biplot), we can state that Compositional HJ-biplot get the best representation of the compositional data, better than “form biplot” and “covariance biplot”, proposed by Aitchison and Grenacre.

To extend to other Biplots, such as MANOVA biplot, the impact on links should be carefully studied. Using this novel statistical technique, linear relationships or links between the variables were identified that are different from those provided by the usual correlation coefficient studies. The value of links allows new ways to study the relationship among variables. In order to validate this statistical methodology, the results were contrasted with other scientific results, however more repetitions in several localities and year are necessary to test the results.

## Figures and Tables

**Table 1:** Proximate and mineral elements composition (mean ± standard deviation, data expressed in fresh weight, FW) of tomato samples grouped according to cultivar and cultivation system.

Compound	Cultivar	Cultivation System		*p*-Value
		Soil	No-Soil	
**Proximate Composition (% FW)**				
Moisture	Dunkan	93.78 ± 0.55	94.11 ± 0.51	0.061 ^1^
	Mariana	94.28 ± 0.47	93.88 ± 0.58	
	*p*-Value	0.129 ^2^		
Ash	Dunkan	0.75 ± 0.09	0.73 ± 0.04	0.159
	Mariana	0.69 ± 0.07	0.74 ± 0.07	
	*p*-Value	0.952		
Protein	Dunkan	0.68 ± 0.05	0.75 ± 0.13	**0.049**
	Mariana	0.70 ± 0.07	0.77 ± 0.07	
	*p*-Value	**0.015**		
Fructose	Dunkan	1.41 ± 0.09	1.38 ± 0.24	0.765
	Mariana	1.24 ± 0.22	1.39 ± 0.17	
	*p*-Value	0.145		
Glucose	Dunkan	1.40 ± 0.09	1.37 ± 0.24	0.798
	Mariana	1.23 ± 0.22	1.38 ± 0.17	
	*p*-Value	0.137		
**Mineral Elements (mg/kg FW)**				
P	Dunkan	255 ± 26.0	280 ± 36.3	0.455
	Mariana	242 ± 20.8	276 ± 32.3	
	*p*-Value	**0.014**		
Na	Dunkan	42.29 ± 5.31	46.23 ± 2.67	0.405
	Mariana	40.66 ± 9.63	53.00 ± 12.7	
	*p*-Value	**0.001**		
K	Dunkan	2654 ± 340	2844 ± 332	0.165
	Mariana	2570 ± 275	2859 ± 214	
	*p*-Value	0.093		
Ca	Dunkan	88.76 ± 8.53	83.00 ± 5.74	0.295
	Mariana	85.65 ± 14.4	82.00 ± 7.67	
	*p*-Value	**0.046**		
Mg	Dunkan	159 ± 13	167 ± 18	**3.05 × 10^−4^**
	Mariana	115 ± 16	133 ± 13	
	*p*-Value	**0.009**		
Fe	Dunkan	3.36 ± 0.65	3.14 ± 0.48	0.109
	Mariana	2.66 ± 0.32	2.98 ± 0.48	
	*p*-Value	0.176		
Cu	Dunkan	0.35 ± 0.18	0.47 ± 0.20	0.623
	Mariana	0.32 ± 0.08	0.33 ± 0.04	
	*p*-Value	0.260		
Zn	Dunkan	0.80 ± 0.18	0.99 ± 0.29	0.146
	Mariana	0.98 ± 0.31	0.96 ± 0.12	
	*p*-Value	0.718		
Mn	Dunkan	1.04 ± 0.12	0.99 ± 0.12	0.053
	Mariana	1.04 ± 0.11	1.02 ± 0.13	
	*p*-Value	**0.009**		

^1^ *p*-Value by tomato cultivar; ^2^ *p*-Value by cultivation system. *p*-Values in bold mean significant differences after clr-transformation according to Mann–Whitney U test.

**Table 2:** Organic acids and antioxidant compounds composition (mean ± standard deviation, data expressed in fresh weight, FW) of tomato samples grouped according to cultivar and cultivation system.

Compound	Cultivar	Cultivation System		*p*-Value
		Soil	No-Soil	
**Organic Acids (mg 100 g^−1^ FW)**				
Citric acid	Dunkan	422 ± 64.7	443 ± 93.4	**0.001** ^1^
	Mariana	541 ± 138	561 ± 131	
	*p*-Value	0.999 ^2^		
Malic acid	Dunkan	25.7 ± 9.87	22.1 ± 4.16	0.732
	Mariana	25.5 ± 9.14	19.9 ± 5.95	
	*p*-Value	0.001		
Ascorbic acid	Dunkan	14.8 ± 0.61	14.4 ± 1.38	**0.009**
	Mariana	15.2 ± 2.04	15.0 ± 1.67	
	*p*-Value	0.087		
Oxalic acid	Dunkan	16.9 ± 6.13	17.3 ± 4.06	0.104
	Mariana	13.2 ± 2.93	14.1 ± 4.43	
	*p*-Value	0.798		
Fumaric acid	Dunkan	5.69 ± 1.437	5.62 ± 1.37	**0.026**
	Mariana	4.33 ± 1.37	4.08 ± 1.09	
	*p*-Value	0.499		
Pyruvic acid	Dunkan	1.40 ± 0.44	1.87 ± 0.71	0.608
	Mariana	1.80 ± 0.89	1.40 ± 0.74	
	*p*-Value	0.079		
**Antioxidant Compounds (mg 100 g^−1^ FW)**				
Total phenols	Dunkan	24.3 ± 3.00	23.5 ± 1.07	0.091
	Mariana	26.3 ± 6.77	23.6 ± 6.81	
	*p*-Value	**0.016**		
Lycopene	Dunkan	2.34 ± 0.12	2.63 ± 0.36	**0.001**
	Mariana	1.73 ± 0.36	1.76 ± 0.40	
	*p*-Value	0.741		
Chlorogenic acid	Dunkan	1.08 ± 0.24	1.09 ± 0.13	0.814
	Mariana	0.996 ± 0.26	1.15 ± 0.42	
	*p*-Value	0.344		
Ferulic acid	Dunkan	0.124 ± 0.03	0.125 ± 0.02	0.685
	Mariana	0.121 ± 0.03	0.130 ± 0.02	
	*p*-Value	0.548		
Caffeic acid	Dunkan	0.115 ± 0.02	0.115 ± 0.05	**0.010**
	Mariana	0.083 ± 0.03	0.093 ± 0.02	
	*p*-Value	0.674		

^1^ *p*-Value by tomato cultivar; ^2^ *p*-Value by cultivation system. *p*-Values in bold mean significant differences after clr-transformation according to Mann–Whitney U test.

**Table 3:** Variation array of functional compounds (The upper triangle contains the log-ratio variances and the lower diagonal contains the log-ratio means).

Xi/Xj	Variance ln(Xi/Xj)								clr-Variances
	K	Mg	Ascorbic	Lycopene	Phenols	Chlorogenic	Caffeic	Ferulic	
K		0.026	0.018	0.063	0.057	0.061	0.121	0.047	0.012
Mg	−30.59		0.045	0.043	0.104	0.087	0.111	0.081	0.025
Ascorbic	−28.77	0.182		0.084	0.051	0.080	0.138	0.057	0.022
Lycopene	−50.01	−19.42	−21.24		0.126	0.105	0.129	0.099	0.044
Phenols	−23.87	0.672	0.490	26.14		0.112	0.164	0.092	0.052
Chlorogenic	−55.68	−25.09	−26.90	−0.567	−31.81		0.089	0.038	0.035
Caffeic	−80.43	−49.84	−51.66	−30.42	−56.56	−24.76		0.130	0.073
Ferulic	−77.02	−46.43	−48.25	−27.01	−53.15	−21.34	0.341		0.031
Mean ln(Xi/Xj)									0.294
